# Characteristics of Fe/C catalysts based on pyrolysis of ferric citrate and its peroxymonosulfate activation performance to degrade sulfadiazine in water†

**DOI:** 10.1039/d4ra00768a

**Published:** 2024-05-14

**Authors:** Baowei Zhao, Jiani Yang

**Affiliations:** a School of Environmental and Municipal Engineering, Lanzhou Jiaotong University No. 88, West Anning Rd Lanzhou 730070 P. R. China zhbw2001@sina.com +86-931-4955760 +86-931-4955760

## Abstract

Advanced oxidation techniques based on peroxysulfate activation have been paid much attention owing to their excellent performance in degrading stubborn pollutants in water. In response to the current situation that requires more raw materials and higher costs and involves more complicated processes for the preparation of Fe/C catalysts to activate persulfates, novel catalysts (Fe/C-700, Fe/C-800, Fe/C-900 and Fe/C-1000) were prepared by a high-temperature carbonization method at different pyrolysis temperatures (700, 800, 900 and 1000 °C) using inexpensive and environmentally friendly ferric citrate as raw material. Fe/C catalysts were characterized using SEM, EDS, XRD, XPS, and VSM and were screened for the activation of peroxymonosulfate (PMS) to degrade sulfadiazine (SDZ) in water, where Fe/C-900 exhibited higher efficiency. Thus, its activation performance for PMS to degrade SDZ was comprehensively investigated and the mechanisms of activation degradation were analyzed. The results showed that the degradation rate of 98.7% can be achieved to 10 mg L^−1^ SDZ by 0.1 g L^−1^ Fe/C-900 and 0.5 mmol L^−1^ PMS within 60 min. A wide range of solution pH, low catalyst dosage and good recycling performance were found in the Fe/C-900 application and the amount of iron ions dissolved at the end of the reaction was low (0.350 mg L^−1^). It was shown that both free radical and non-free radical pathways existed in the reaction system, where ^1^O_2_, SO_4_^−^˙ and O_2_^−^˙ played dominant roles in the degradation process of SDZ. The results could provide new ideas for the preparation of Fe/C catalysts and their heterogeneous activation for PMS to degrade stubborn organic pollutants in water.

## Abstract 2

Advanced oxidation techniques based on peroxysulfate activation have been paid much attention owing to their excellent performance in degrading stubborn pollutants in water.

## Graphical abstract

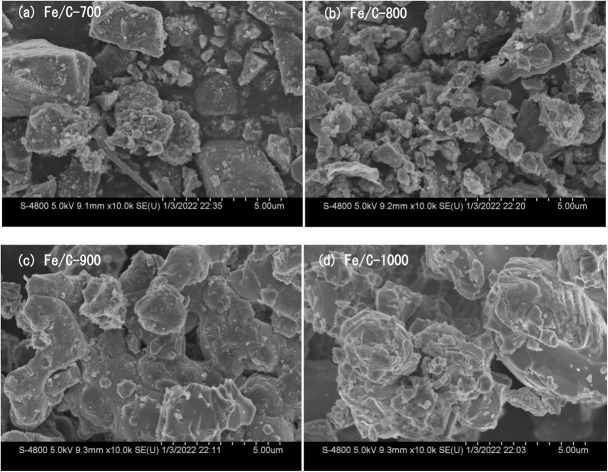

## 1 Introduction

Antibiotics are widely used in human and veterinary medicine due to their lower cost and higher effectiveness. However, misuse and improper handling have led to their widespread detection in aquatic environments.^1,2^ Although antibiotics present in water media mostly at low concentrations, their sources of contamination are widespread,^3,4^ and traditional treatment methods are difficult to remove them effectively,^5^ thus posing a serious threat to human health and the ecological environment. Therefore, it is necessary to seek an efficient and environmentally friendly method for the treatment of wastewater containing antibiotics, *e.g.* sulfonamide.

Novel advanced oxidation techniques based on peroxysulfate activation (SR-AOPs) have been widely studied and noticed due to their excellent performance in degrading stubborn pollutants.^6^ Compared to the oxidative activity potential of hydroxyl radicals (OH˙) (1.9–2.7 V) generated by the commonly used conventional Fenton reagents, the sulfate radicals (SO_4_^−^˙) generated by activating peroxysulfate have higher oxidative activity potential (2.6–3.1 V), together with a longer half-life (OH˙: <1 μs, SO_4_^−^˙: 30–40 μs).^7^ Moreover, it has better activity for wastewater treatment within a wide range of pH values and is inherently more stable.^8^ It is easy to store, transport and apply to treat wastewater containing hard-to-degrade organic compounds (*e.g.* antibiotics).^9^ In the SR-AOP system, the activation method of persulfate directly determines the rate of SO_4_^−^˙ production and the effectiveness of degrading pollutants, so it is crucial to choose the appropriate activation method. Currently, heterogeneous catalysts such as metal oxides, carbon materials, and metal-loaded catalysts are widely used in the processes, among which Fe/C catalysts exhibited a priority in activation, separation, and recovery and avoiding serious secondary contamination by transition metal dissolution.^10,11^ However, the current situation (*e.g.* Fe-loaded biochars) requires more raw materials and higher costs and involves more complicated processes for the preparation of Fe/C catalysts, limiting the application of Fe/C catalysts.

Thus, Fe/C catalysts (Fe/C-700, Fe/C-800, Fe/C-900 and Fe/C-1000) were prepared under different pyrolysis temperatures (700, 800, 900 and 1000 °C) by high-temperature carbonization methods using ferric citrate, which is green and non-toxic and contains both Fe and C elements. The chemical composition and morphological structure of the catalysts were analyzed. The effects of pyrolysis temperature on their activation performance for peroxymonosulfate (PMS) were studied using sulfadiazine (SDZ) as one of the typical antibiotics. Then, the comprehensive activation performance and mechanisms in the degradation of SDZ in water by PMS were explored, with a view to providing new ideas for the preparation of Fe/C catalyst and treatment of refractory organic pollutants by advanced oxidation technology based on persulfate.

## 2 Experimental

### 2.1 Materials and reagents

Sulfadiazine (SDZ), ferric citrate, *p*-benzoquinone and l-histidine were analytically pure and purchased from Shanghai Maclean Biochemical Technology Co., China. Methanol (MeOH), *tert*-butanol, hydrochloric acid, sodium hydroxide, potassium peroxymonosulfate (PMS) and anhydrous ethanol were analytically pure, and acetonitrile was chromatographically pure, purchased from Tianjin Damao Chemical Reagent Co., China. Water used for the mobile phase in liquid chromatography was ultrapure water, and the experimental water was deionized.

### 2.2 Synthesis of catalysts

The appropriate amount of ferric citrate was weighed and placed flat in a quartz boat, which was transferred into a tube furnace (OTF-1200X, Hefei Kejing Materials Technology Co., Ltd, China), and the air in the tube was evacuated by passing N_2_ at a constant rate. The pyrolysis was set up at 700, 800, 900 and 1000 °C at a rate of 5 °C min^−1^ and 2 h of heating time. Then, the products were taken out and sieved through a 200-mesh sieve. The obtained Fe/C catalysts were placed into the sealed bags, labeled as Fe/C-700, Fe/C-800, Fe/C-900 and Fe/C-1000 and stored in a vacuum dryer (DZF-6012, Shanghai Yiheng Scientific Instruments Co., Ltd, China).

### 2.3 Characterization of catalysts

The microstructure and surface characteristics of the samples were observed by field emission scanning electron microscopy (SEM) (JSM-6700F, Jie-Olu Company, Japan); the elemental content of the sample surface was analyzed by X-ray energy spectrometry (EDS) (ESCALAB 250, Thermo Scientific, Shanghai, China); the specific surface area and pore size structure of the samples were determined and analyzed on a specific surface area and pore size analyzer (BET) (ASAP2010, Micromeritics, USA); the crystal structure characteristics of the samples were analyzed by X-ray diffractometer (XRD) (SmartLab, Rigaku Company, Japan); the composition and chemical forms of the elements on the sample surface were detected by X-ray photoelectron spectroscopy (XPS); a vibrating sample magnetometer (VSM) (Lakeshore-7404, Lake Shore Co., USA) was used to determine the magnetic strength of the catalyst samples.

### 2.4 Experimental methods

The experiments were carried out in 150 mL conical flasks, which were placed in a constant temperature gas bath shaker (THZ-82A, Jiangsu Danyangmen Quartz Glass Factory, China) at 25 °C and 200 rpm for the reaction. To screen the prior catalyst (*i.e.* effect of the pyrolysis temperature on the catalytic efficiency), 100 mL of the prepared SDZ solution was added to the conical flask, and the solution pH value was adjusted to the set value with 0.1 mol L^−1^ HCl and NaOH solutions. The catalyst was added and the SDZ adsorption reaction was performed for 60 min, and then PMS was added to initiate the degradation reaction for 60 min, where initial SDZ concentration = 40 mg L^−1^, PMS concentration = 0.5 mmol L^−1^, Fe/C dosage = 0.2 g L^−1^, pH = 7.0 and reaction time = 60 min. To compare the effects of ferric citrate alone, Fe/C-900 alone, PMS alone and PMS with Fe/C-900 (Fe/C-900 + PMS) on the degradation of SDZ, the initial conditions were kept as SDZ concentration = 10 mg L^−1^, PMS concentration = 1.0 mmol L^−1^, ferric citrate or Fe/C dosage = 0.05 g L^−1^, pH = 7.0 and reaction time = 60 min. As for the comprehensive activation performance of Fe/C-900, no adsorption period was set up, where a single factor method was used to test the effects of Fe/C-900 dosage (0.05, 0.10, 0.15 and 0.20 g L^−1^), PMS concentration (0.1, 0.3, 0.5 and 1.0 mmol L^−1^), pH value (3, 5, 7 and 9), initial SDZ concentration (5.0, 10, 20 and 40 mg L^−1^), co-existing anions (5 mmol L^−1^ Cl^−^ or 5 mmol L^−1^ HCO_3_^−^) and 5.0 mg L^−1^ humic acid (HA) on the degradation of SDZ.

At certain time intervals, 1 mL of the reaction sample was collected and transferred into a colorimetric tube containing methanol to terminate the reaction, followed by passing a 0.22 μm PTFE membrane, and the SDZ concentration at each moment was quantified by high-performance liquid chromatography (HPLC). The residual iron ions were determined. All experiments were repeated three times, and the average value was taken as the analytical results.

To evaluate the contribution of the corresponding active species to the catalytic degradation of SDZ, *tert*-butanol was selected as a quencher of OH˙,^12^ MeOH was selected as a quencher of SO_4_^−^˙and OH˙,^13^ and benzoquinone was selected as a quencher of O_2_^−^˙to identify the effect of the free radical pathway in the system.^14^ To investigate the presence of non-radical pathways in the system, l-histidine (L-HIS) was selected as a quencher for ^1^O_2_.^15^ The residual iron ions were determined.

### 2.5 Analytical methods

SDZ concentration (*C*_*t*_) at a reaction time (*t*) was determined on a high-performance liquid chromatograph (LCQ Advantage 4000, Thermo Scientific, USA). The operation conditions were as follows: a C18 reversed-phase column (4.6 × 250 mm, 5 μm), detection wavelength 269 nm, column temperature 30 °C, mobile phase of acetonitrile and water (25 : 75, v/v), flow rate 1 mL min^−1^, and injection volume 20 μL. The ratio *C*_*t*_/*C*_0_ of SDZ was used as an index to evaluate the degradation performance. The residual iron ions were determined on a UV-visible spectrophotometer (Shanghai Precision Scientific Instruments Co., Ltd, China) using the *o*-phenanthroline method.

## 3 Results and discussion

### 3.1 Characterization of Fe/C catalysts

**Fig. 1:**
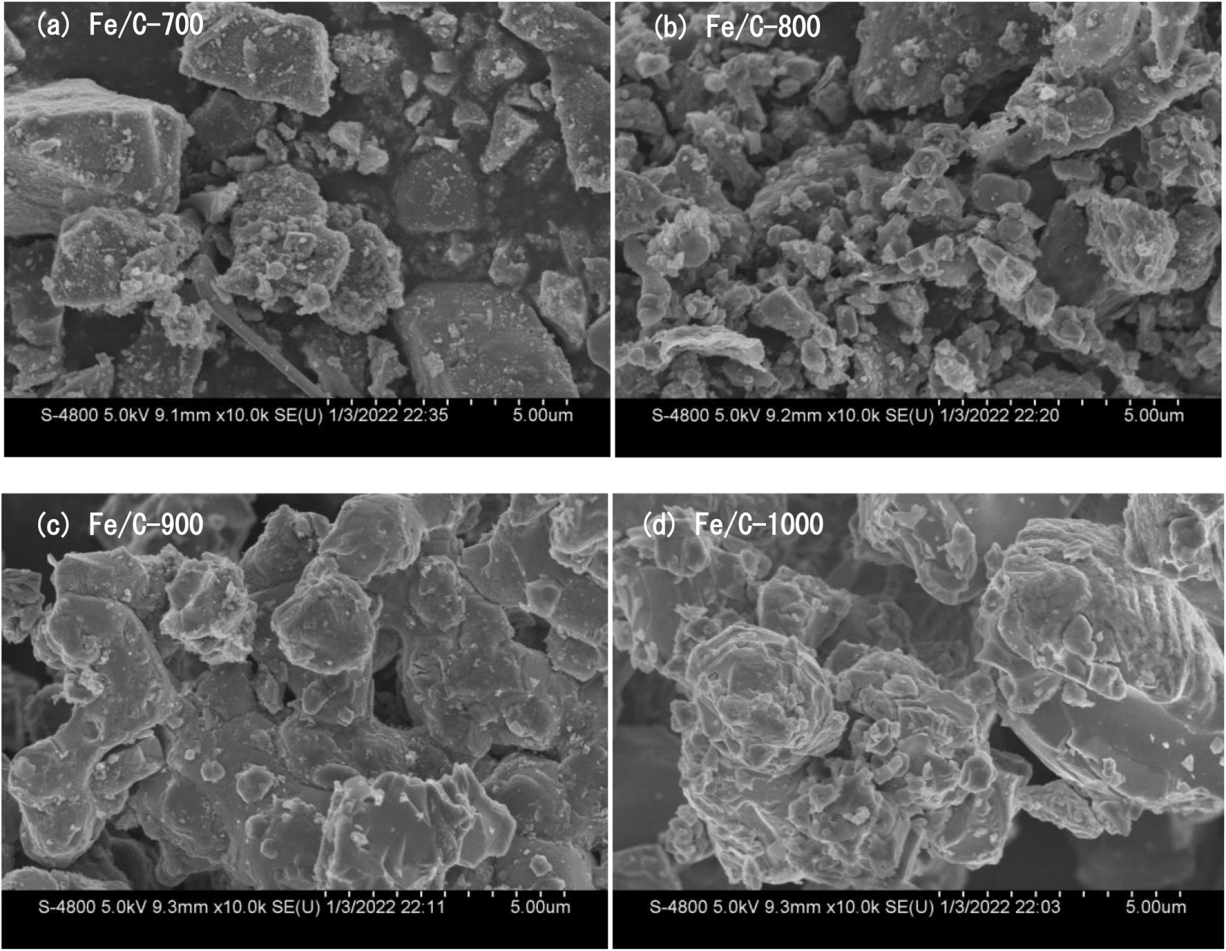
SEM images of Fe/C catalysts.

The microscopic morphology and structure of Fe/C catalysts were observed by scanning electron microscopy (Fig. 1). The figures show that different pyrolysis temperatures corresponded to different morphological structures.^16^ Some granular bumps appeared, which were presumed to be encapsulated FeO nanoparticles in combination with XRD characterization.^17^ As the pyrolysis temperature increased to 900 °C and 1000 °C, some pores collapsed and particle agglomeration was observed on Fe/C-900 and Fe/C-1000 (Fig. 1c and d), which may be due to the excessive pyrolysis temperature.^18^

The surface elemental content in the Fe/C catalysts was analyzed by EDS energy spectroscopy and the related results are presented in Table 1. It can be seen that the surface elemental contents of Fe/C catalysts varied significantly. With the increase of pyrolysis temperature from 700 °C to 800 °C, the surface contents of C and O elements decreased, while the surface content of Fe elements increased, which may be attributed to the fact that a large amount of C and O in ferric citrate gradually underwent redox reactions with Fe, producing Fe in various valence states. However, with the temperature increasing further (900 °C to 1000 °C), the contents of Fe decreased and approached stable values at large.

**Table 1:** Surface element content of Fe/C catalysts

Catalyst	C		O		Fe	
	Wt/%	At/%	Wt/%	At/%	Wt/%	At/%
Fe/C-700	21.73	45.21	17.58	27.56	60.79	27.23
Fe/C-800	7.180	24.31	4.590	11.66	88.23	64.03
Fe/C-900	4.190	13.05	13.80	32.23	82.01	54.72
Fe/C-1000	3.030	9.650	14.14	33.80	82.83	56.55

**Fig. 2:**
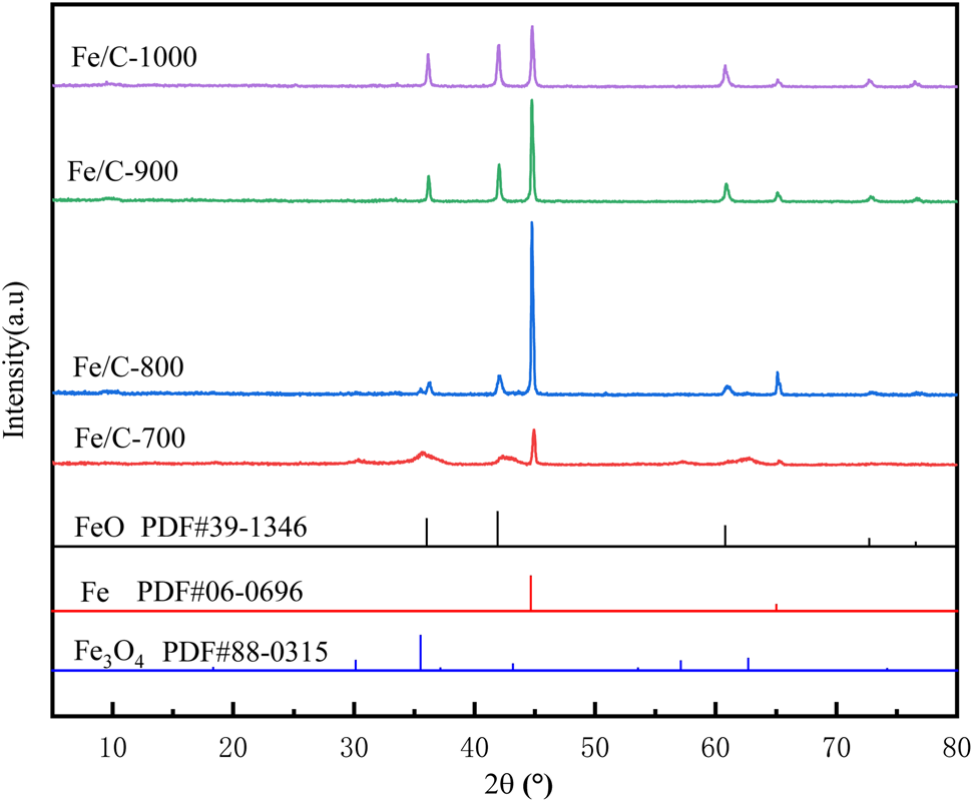
XRD patterns of Fe/C catalysts.

Fig. 2 shows the XRD patterns of Fe/C catalysts. It can be seen that the diffraction peaks of Fe_3_O_4_ (PDF#88-0315) and Fe (PDF#06-0696) appeared in the pattern of the Fe/C-700 sample, indicating that a certain amount of Fe_3_O_4_ and a small amount of Fe were generated at this time, and the production of Fe should be due to the reduction of Fe^2+^ and Fe^3+^ by reducing substances such as CO produced by high-temperature pyrolysis.^19^ When the temperature continues to increase to 800 °C, the diffraction peak of Fe in the pattern of the Fe/C-800 catalyst is obviously enhanced, and it can be seen that the diffraction peak of Fe_3_O_4_ is replaced by FeO (PDF#39-1346), indicating that the Fe_3_O_4_ in the sample is further reduced by C to FeO and Fe. With the increase of temperature to 900 and 1000 °C, the diffraction peak of FeO has a small enhancement and the diffraction peak of Fe gradually becomes weaker, and a part of Fe in the sample is oxidized to FeO at this time. The pyrolysis temperature has a great influence on the crystalline phase composition of the products obtained from high-temperature ferric carbonate and different valence states of iron oxides and carbon forms are formed at different temperatures.

**Fig. 3:**
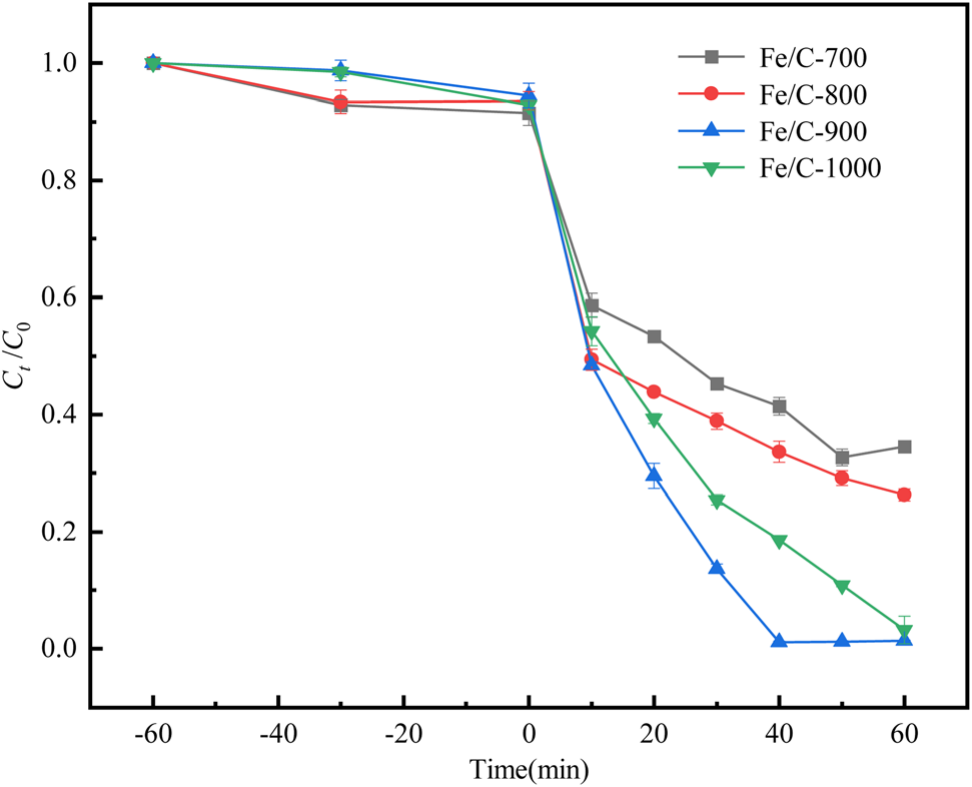
Removal of SDZ by Fe/C catalysts without PMS addition and degradation of SDZ by PMS activated with Fe/C catalysts.

XPS was used to further analyze the elemental composition and valence state of Fe/C catalysts. Fig. S1† shows that all are composed of C, Fe, and O elements. From the high-resolution spectra, the characteristic peaks of C1s spectrum are at 284.8 eV, 285.8 eV, and 288.6 eV, corresponding to the three bonds of C–C, C–O, and C=O, respectively; three characteristic peaks exist in the O1s spectrum: Fe–O (530.4 eV), C=O (532.5 eV), and C–O (533.6 eV) indicating the presence of iron oxides in the catalysts, which is consistent with the XRD characterization; for Fe2p, the characteristic peaks of Fe2p_3/2_: Fe^2+^ (710 eV), Fe^3+^ (712 eV) and Fe2p_1/2_: Fe^2+^ (724 eV), Fe^3+^ (725 eV) were observed at 711.4 eV and 724.6 eV, respectively. The abundance of Fe^2+^ and Fe^3+^, mainly Fe^2+^, will promote the system to activate PMS efficiently and continuously. As can be seen from Fig. S1d,† the catalysts at different temperatures have different compositions of iron oxidation states. Fe/C-1000 possesses the highest Fe^2+^ percentage and Fe/C-800 possesses the highest Fe^0^ percentage. For Fe^2+^ and Fe^0^, two important species in the catalytic system, Fe/C-900 has a high content of both of them (Fe^2+^: 58.4%, Fe^0^: 11.8%), although one is not the highest when viewed separately. The abundance of active substances, coupled with a balanced and excellent substance content ratio will undoubtedly contribute to the catalytic degradation of SDZ by the system, which is also consistent with the catalytic performance experiments described in the later section (Fig. 3). All of the Fe/C catalysts showed peaks near 708 eV. In addition, the characteristic peaks of Fe^0^ appear around 708 eV in all the Fe/C catalysts, indicating the presence of a certain amount of zero-valent iron in them.^20^ In conclusion, Fe/C catalysts contain rich oxygen-containing functional groups and carbon-unsaturated bonds to transfer electrons to PMS to produce more radicals, which could promote its interaction with SDZ (see the following Sections 3.3–3.5). In addition, a large number of iron oxides are generated, among which there is Fe^0^,^20^ which can significantly improve the catalytic effect to efficiently cooperate with the catalytic system.

### 3.2 Effect of catalyst pyrolysis temperature on SDZ degradation

Fig. 3 shows *C*_*t*_/*C*_0_ of SDZ *versus* the reaction time *t* for Fe/C-700, Fe/C-800, Fe/C-900 and Fe/C-1000 to activate PMS to degrade SDZ, considering the adsorption process without the addition of PMS before degradation. The adsorptive removal of SDZ by Fe/C catalysts was less than 10% up to 60 min of adsorption, which is related to the small specific surface area of all four catalysts in the BET characterization (Fe/C-700, 67.4; Fe/C-800, 76.8; Fe/C-900, 2.49; and Fe/C-1000, 0.637 m^2^ g^−1^) and is in significant contrast to the degradation removal of SDZ initiated by the addition of PMS oxidant (*t* = 0 min) in the latter 60 min.

Fe/C-700, Fe/C-800, Fe/C-900 and Fe/C-1000 showed different catalytic abilities in activating PMS to degrade SDZ. When the pyrolysis temperature was increased from 700 to 800 °C, the degradation rates of SDZ increased from 65.5% to 73.7%, and Fe/C-900 corresponded to a 98.6% SDZ degradation rate, while the degradation rates of SDZ by Fe/C-1000 decreased slightly to 96.8% when the pyrolysis temperature was increased to 1000 °C. Therefore, Fe/C-900 was selected as the target catalyst to activate PMS to degrade SDZ for further study. The better performance of Fe/C-900 is also consistent with its high content of Fe^2+^: 58.4% and Fe^0^: 11.8%, as mentioned above.

### 3.3 Comparison of the removal of SDZ under different reaction systems

**Fig. 4:**
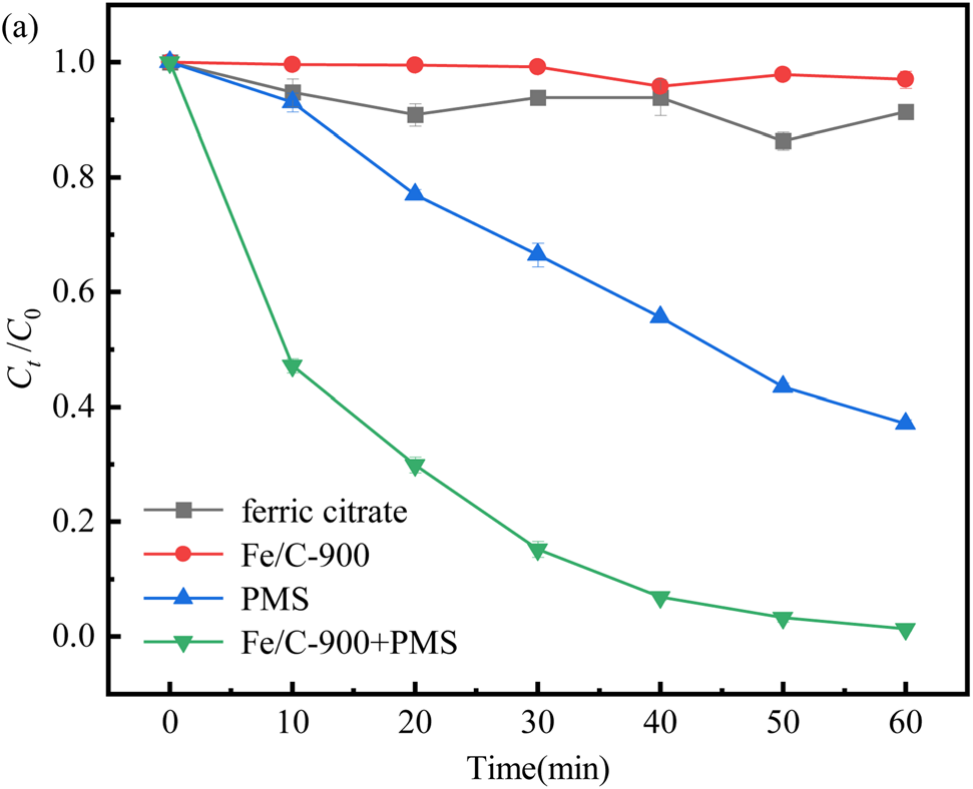
Comparison of the removal of SDZ by ferric acid, Fe/C-900, PMS and Fe/C-900 with PMS.

The variations of SDZ removal by ferric acid, Fe/C-900, PMS and Fe/C-900 with PMS with the reaction time are shown in Fig. 4, respectively. It can be seen that after 60 min of the reaction, the adsorption removal of SDZ by ferric citrate and Fe/C-900 alone was very low and basically negligible. When only the oxidant PMS was present in the SDZ solution, it contributed 62.9% to the oxidative removal of SDZ, which indicated that PMS alone has a higher ability to remove SDZ by oxidation. This is similar to the results of Chen and Yang *et al.*,^21,22^ who stated that PMS can directly and efficiently oxidatively degrade a variety of organic pollutants such as phenols and antibiotics in the absence of activation through the intermolecular peroxide bond breakage, *etc.* It was shown that the direct oxidative removal of organic pollutants by PMS also has some application potential. However, when the oxidant PMS and catalyst Fe/C-900 were simultaneously added to the system, the degradation and removal of SDZ were significantly enhanced, and basically, the 10 mg L^−1^ SDZ was completely degraded, which was attributed to the iron ions and abundant functional groups contained in Fe/C-900. On the one hand, they effectively promoted the production of SO_4_^−^˙ and other strong oxidizing substances. On the other hand, Fe and C in Fe/C-900 act synergistically in the reaction system and can provide a source for stable and continuous release of Fe^2+^.

### 3.4 Activation performance of Fe/C-900 for PMS to degrade SDZ

**Fig. 5:**
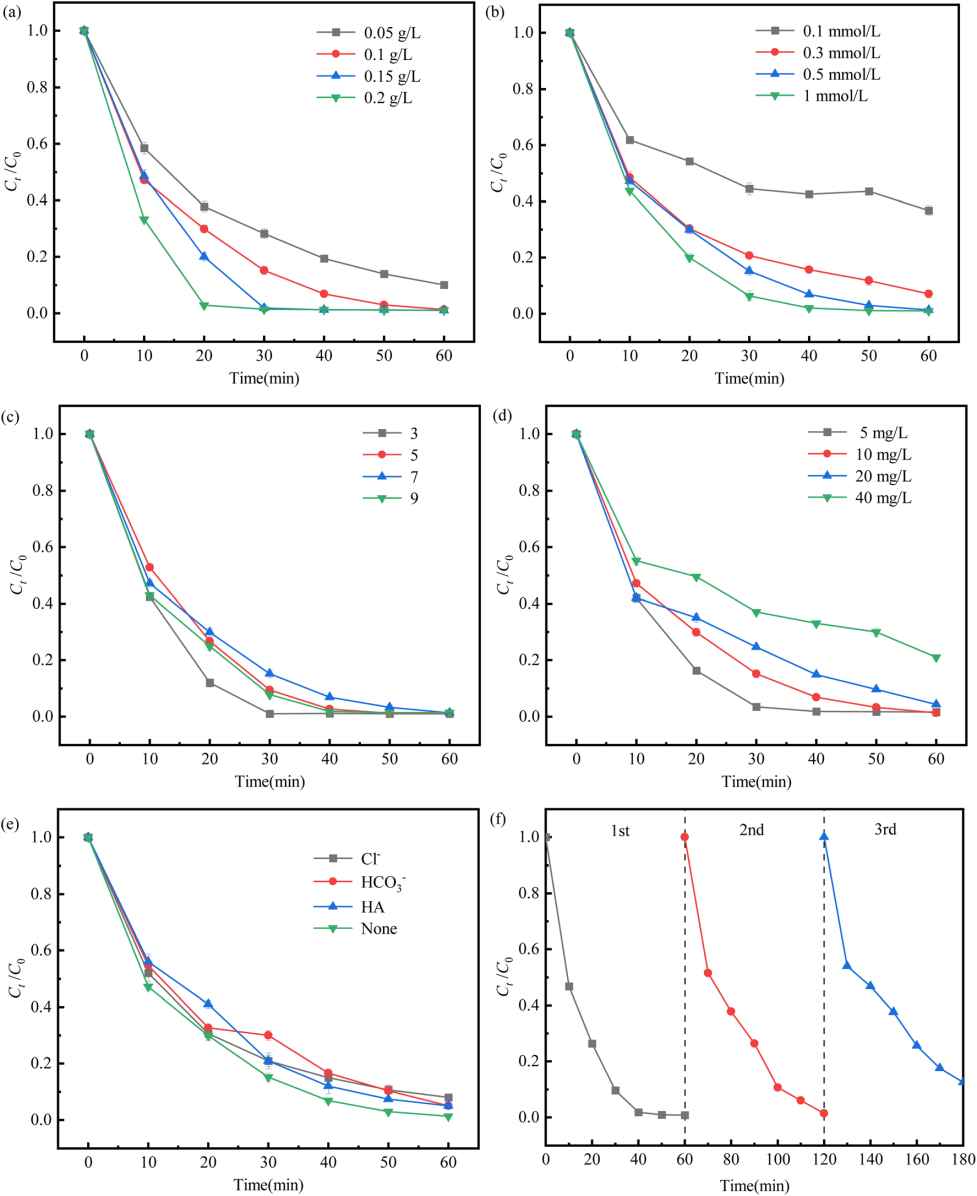
Activation performance of Fe/C-900 for PMS to degrade SDZ. (a) Fe/C-900 dosage; (b) PMS concentration; (c) initial pH value; (d) initial SDZ concentration; (e) co-existing anions and HA; and (f) Fe/C-900 recycling use.

The activation performance of Fe/C-900 for PMS to degrade SDZ is shown in Fig. 5 when Fe/C-900 dosage, PMS concentration, initial pH value, initial SDZ concentration, and co-existing anions and HA were considered, together with the recycling use of Fe/C-900. As shown in Fig. 5a, a small dosage of Fe/C-900 cannot achieve the expected activation efficiency, and a higher dosage will lead to a waste of resources. The degradation rates of SDZ by Fe/C-900 at the dosages of 0.05, 0.1, 0.15, and 0.2 g L^−1^ were 89.9%, 98.7%, 99.0%, and 98.9%, respectively. The degradation rate increased with the increase of Fe/C-900 dosage, but it showed a decreasing trend at the dosage of 0.2 g L^−1^. Thus, 0.1 g L^−1^ of Fe/C-900 dosage was selected to maximize its activation capacity and activation capacity.

As an important factor in the degradation of pollutants in the persulfate system, the dosage of PMS has an important influence on the degradation of pollutants. As Fig. 5b shows, 0.1, 0.3, 0.5 and 1 mmol L^−1^ of PMS lead to 63.2%, 92.9%, 98.7% and 99.0% of SDZ degradation, respectively. In the degradation of SDZ by PMS activated with Fe/C-900, the generation of strong oxidizing SO_4_^−^˙ depends on the activation and release of PMS. Therefore, moderately increasing the PMS dosage will improve the SDZ degradation rate and reaction rate when other experimental conditions remain unchanged; meanwhile, at a higher PMS dosage, the rate of its corrosion of Fe^0^ and the ability to release Fe^2+^ and Fe^3+^ will be enhanced.^23^ However, quenching reactions of free radicals with free radicals, and free radicals with PMS^24,25^ will occur when there is too much of PMS, reducing the reaction rate. Thus, 0.5 mmol L^−1^ of PMS concentration was chosen for the following steps.

The effects of initial solution pH values, 3, 5, 7 and 9, are shown in Fig. 5c. It can be seen that the Fe/C-900 catalyst maintained better SDZ degradation rates within the pH range of 3 to 9, which proves that the system was stable over a wide range of pH values. The SDZ degradation rate was 99.0% at pH 3 and slightly decreased to 98.7% and 98.6% at pH 7 and 9, respectively. The reduction of H^+^ under alkaline conditions slowed down the corrosion rate of iron in the system, while the decomposition reaction of PMS also weakened its ability to release active substances.^26,27^ In order to maintain a high catalytic activity in the system and to reduce the leaching of Fe ions under acidic conditions, the experiments were chosen to be carried out under neutral conditions at pH 7.

Fig. 5d shows the effects of 5.0, 10, 20 and 40 mg L^−1^ of the initial SDZ concentrations on its degradation. It is obvious that the degradation rate decreased continuously as the initial concentration of SDZ increased. This is because the amount of Fe/C-900 and PMS in the system is limited when other conditions are given. The higher the concentration of SDZ, the more intense the competition between the active substance and the active site on the catalyst, which is not conducive to the activation of PMS; too much SDZ will also produce more intermediate products covering a part of the active site, further limiting the degradation of SDZ and inhibiting the increase of the degradation rate.

Fig. 5e shows that co-existing anions (Cl^−^ and HCO_3_^−^) and HA inhibited the SDZ degradation to a certain extent, and the inhibition intensity followed the order of Cl^−^ > HCO_3_^−^ ∼ HA. This indicates that the degradation and removal of SDZ by PMS activated with Fe/C-900 does not mainly depend on the free radical pathway, also including the non-free radical pathway.

The saturation magnetization rate of Fe/C-900 was about 117.0 emu g^−1^, proving that Fe/C-900 could be recycled by magnetic separation. Fe/C-900 was magnetically recycled three times and the results are shown in Fig. 5f, and the degradation rates of SDZ were 99.2%, 98.6% and 87.5%, respectively. The decreasing degradation rate of SDZ is attributed to the gradual destruction of the structure of Fe/C-900 during the reuse process, while some untreated SDZ and its degradation products would remain during the recovery and cleaning process, limiting the ability to continue activating PMS.

### 3.5 Mechanisms on activation of Fe/C-900 for PMS to degrade SDZ

**Fig. 6:**
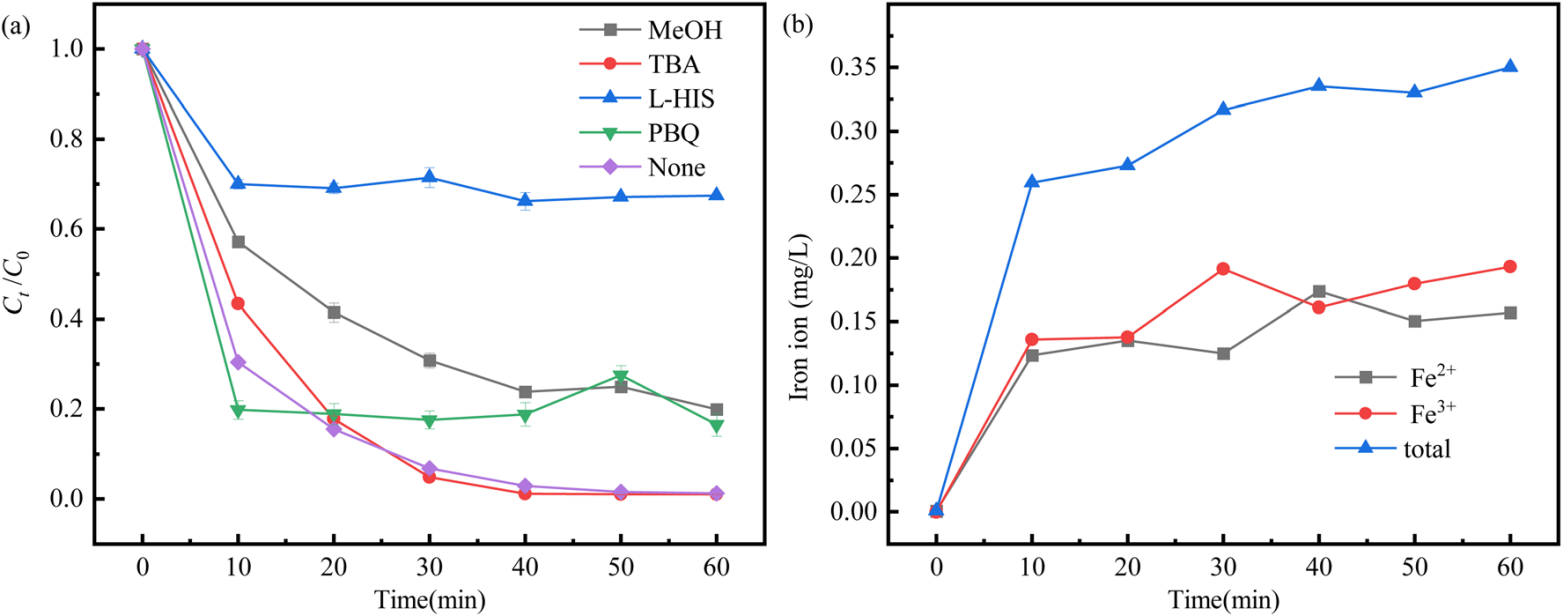
Results of quenching experiments (a) and variation of iron ion concentration (b).

Quenching experiments showed that free radical pathways and non-free radical pathways are acting together for the degradation and removal of SDZ (Fig. 6a). The degree of contribution of each reactive substance in the Fe/C-900 activated PMS system is in the order of ^1^O_2_ > SO_4_^−^˙ >O_2_^−^˙ > OH˙. The processes are mainly non-radical-dominated degradation ones based on ^1^O_2_, and the dominant reactive species is ^1^O_2_. However, the free radical pathway also plays a role in the degradation of SDZ, where the contribution of SO_4_^−^˙ is relatively significant.

Fig. 6b shows the variation of concentrations of Fe^3+^, Fe^2+^ and total iron ions in the SDZ degradation at optimum conditions. At the beginning of the reaction, the concentration of Fe^2+^ in the system showed a rising trend and then decreased, which was due to the fact that after the reaction was started, Fe/C-900 corroded and released a certain amount of Fe^2+^ into the reaction system to activate PMS to degrade SDZ, and after a period of degradation, the Fe^2+^ consumption was reduced and converted to Fe^3+^. In the middle and late stages of the reaction, the presence of C and Fe^0^ in the system reduced Fe^3+^ to Fe^2+^, so there was a tendency for the Fe^3+^ concentration to decrease and the Fe^2+^ concentration to increase twice. During the 60 minutes reaction, Fe^3+^ was mostly more than Fe^2+^ in the system solution, which also indicated that it was Fe^2+^ that was mainly involved in the activation reaction. In addition, the total iron ions dissolved in the system at the end of the reaction were little, 0.350 mg L^−1^, which is in accordance with the relevant emission standards.

**Fig. 7:**
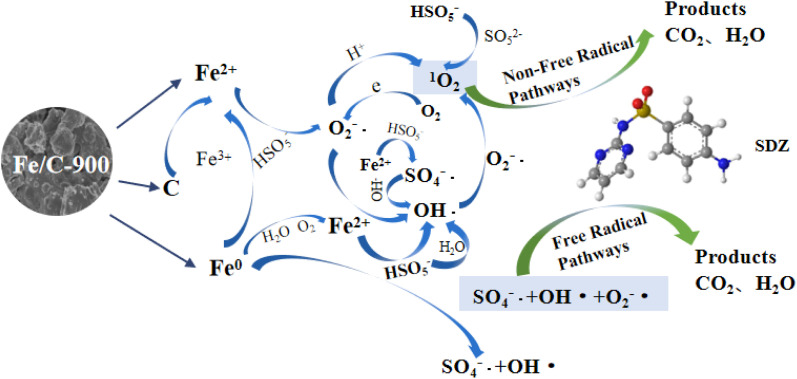
Mechanisms on activation of Fe/C-900 for PMS to degrade SDZ.

In view of the results above, the mechanism of the activation of Fe/C-900 for PMS to degrade SDZ was presumed and shown in Fig. 7. The reaction processes might involve the following steps: (1) the iron component in the Fe/C-900 is converted from Fe^2+^ to Fe^3+^, and at the same time, electrons are released to activate PMS to break the peroxide bond to produce SO_4_^−^˙ and OH˙ (eqn (1)), and PMS is hydrolyzed to produce H_2_O_2_, which then decomposes to produce OH˙ (eqn (2) and (3)). (2) Fe^0^ can directly activate PMS to produce SO_4_^−^˙ and OH˙ (eqn (4) and (5)), and the ensuing Fe^2+^ can further enhance the generation of free radicals and cause SDZ degradation according to the reactions in step (1); Fe^0^ can also serve as the source of Fe^2+^ in the system and release Fe^2+^ continuously and slowly (eqn (6) and (7)); Fe^0^ and C act as strong reducing components and can also reduce Fe^3+^ to Fe^2+^ (eqn (8) and (9)), which is conducive to the cyclic conversion of active substances and SDZ degradation and is also the reason for the low dissolution of iron ions in the system. (3) Fe/C-900 will also promote the hydrolysis of PMS to generate O_2_^−^˙ (eqn (10) and (11));^28^ the XPS characterization results demonstrate the presence of Fe^2+^, Fe^3+^, and Fe^0^ in Fe/C-900, and the mixed valence state represents the presence of oxygen vacancies in Fe/C-900 composites or defect sites, which would contribute to electron transfer, allowing O_2_ to gain electrons from oxygen vacancies for the conversion to O_2_^−^˙ (eqn (12)).^29^ (4) ^1^O_2_ in the non-radical pathway is the dominant active species in the catalytic system and can be produced through O_2_^−^˙ and OH˙ (eqn (13) and (14)), while the decomposition of PMS itself can also produce ^1^O_2_ (eqn (15)),^30^ and the presence of Fe/C-900 in turn accelerates this process. (5) The radical-active substances SO_4_^−^˙, OH˙, O_2_^−^˙, and the non-radical-active substance ^1^O_2_ act together in the degradation process of SDZ (eqn (16)).

1 Fe^2+^ + 2HSO_5_^−^ → Fe^3+^ + SO_4_^−^˙+ 2OH˙ + SO_4_^2−^

2 HSO_5_^−^ + H_2_O → HSO_4_^−^ + H_2_O_2_

3 H_2_O_2_ → 2OH˙

4 Fe^0^ + 2HSO_5_^−^ → Fe^2+^ + 2SO_4_^−^˙+2OH^−^

5 Fe^0^ + 3HSO_5_^−^ → Fe^3+^ + 3SO_4_^2−^ + 3OH˙

6 2Fe^0^ + 2H_2_O + O_2_ → 2Fe^2+^ + 4OH^−^

7 Fe^0^ + 2H_2_O → Fe^2+^ + 2OH^−^ +H_2_

8 Fe^0^ + 2Fe^3+^ → 3Fe^2 +^

9 C + 4Fe^3+^ + 4OH^−^ → 4Fe^2+^ + CO_2_ + 2H_2_O

10 HSO_5_^−^ → SO_5_^2−^ + H^+^

11 SO_5_^2−^ + H_2_O → O_2_^−^˙ + SO_4_^2−^ + 2H ^+^

12 O_2_ + e^−^ → O_2_^−^˙

13 O_2_^−^˙ + OH˙ → ^1^O_2_ + OH^−^

14 2O_2_^−^˙ + 2H^+^ → H_2_O_2_ + ^1^O_2_

15 HSO_5_^−^ + SO_5_^2−^ → SO_4_^2−^ + HSO_4_^−^ + ^1^O_2_

16 SO_4_^−^˙/OH˙/O_2_^−^˙/^1^O_2_ + SDZ → SDZ_products_ + CO_2_ + H_2_O

## 4 Conclusions

Fe/C-900 exhibited a good activation performance for PMS to degrade SDZ. Too high or too low Fe/C-900 dosage and high PMS concentrations were not conducive to the continued improvement of the degradation efficiency. The system worked well within a wide range of solution pH values (3–9). When the initial concentrations of SDZ were 5–40 mg L^−1^, high removal efficiencies of SDZ were obtained, indicating the system had the potential to treat multiple types of antibiotic wastewater. Fe/C-900 could be magnetically recycled and reused three times with high activation performance. The inhibition of SDZ degradation by coexisting substances (Cl^−^, HCO_3_^−^, HA) was not significant. The radical-active species, SO_4_^−^˙, OH˙, and O_2_^−^˙, and the non-radical-active species^1^O_2_ acted together to cause degradation of SDZ.

## Author contributions

Baowei Zhao: conceptualization, formal analysis, fund acquisition, project administration, resources, supervision, and writing-review and editing. Jiani Yang: data curation, formal analysis, investigation, methodology, software, validation, visualization, and writing-original draft.

## Conflicts of interest

There are no conflicts of interest to declare.

## Supplementary Material

RA-014-D4RA00768A-s001
