# Supplementary material for: Characteristics of Fe/C catalysts based on pyrolysis of ferric citrate and its peroxymonosulfate activation performance to degrade sulfadiazine in water
Source: RSC Adv. 2024 May 14;14(22):15582–90. doi: 10.1039/d4ra00768a (PMC11091591; doi:10.1039/d4ra00768a)
Supplement: RA-014-D4RA00768A-s001 [file RA-014-D4RA00768A-s001.pdf]

Electronic Supplementary Material (ESI) for RSC Advances.
This journal is © The Royal Society of Chemistry 2024

Supplementary data

Manuscript ID: RA-ART-01-2024-000768.R1

TITLE: Characteristics of Fe/C catalysts based on pyrolysis of ferric citrate and its activation performance for peroxymonosulfate to degrade sulfadiazine in water

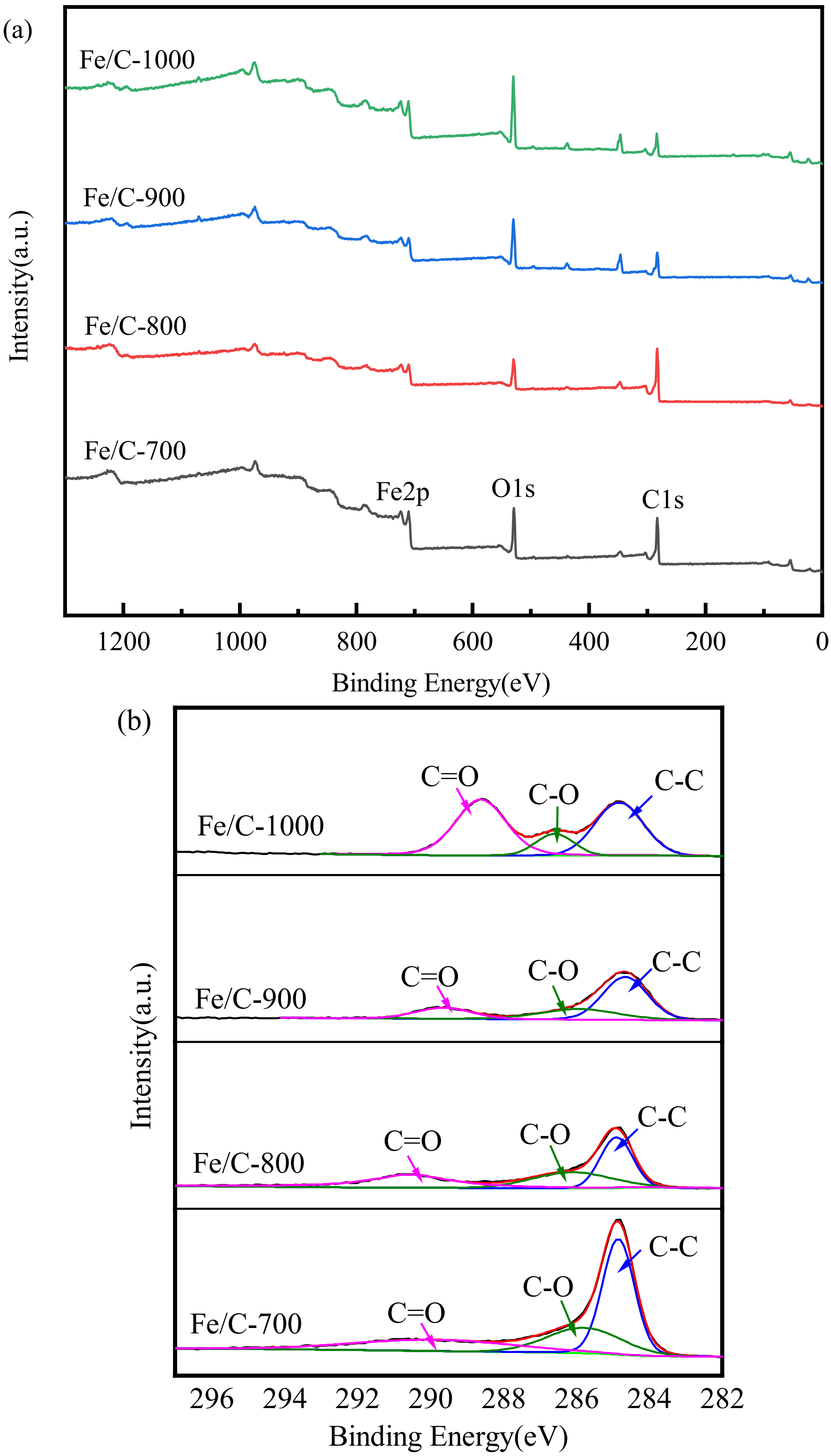

(a)
Fe/C-1000
Fe/C-900
Fe/C-800
Fe/C-700
Fe2p
O1s
C1s
Intensity(a.u.)
1200
1000
800
600
400
200
0
Binding Energy(eV)
(b)
Fe/C-1000
Fe/C-900
Fe/C-800
Fe/C-700
C=O
C-O
C-C
Intensity(a.u.)
296
294
292
290
288
286
284
282
Binding Energy(eV)

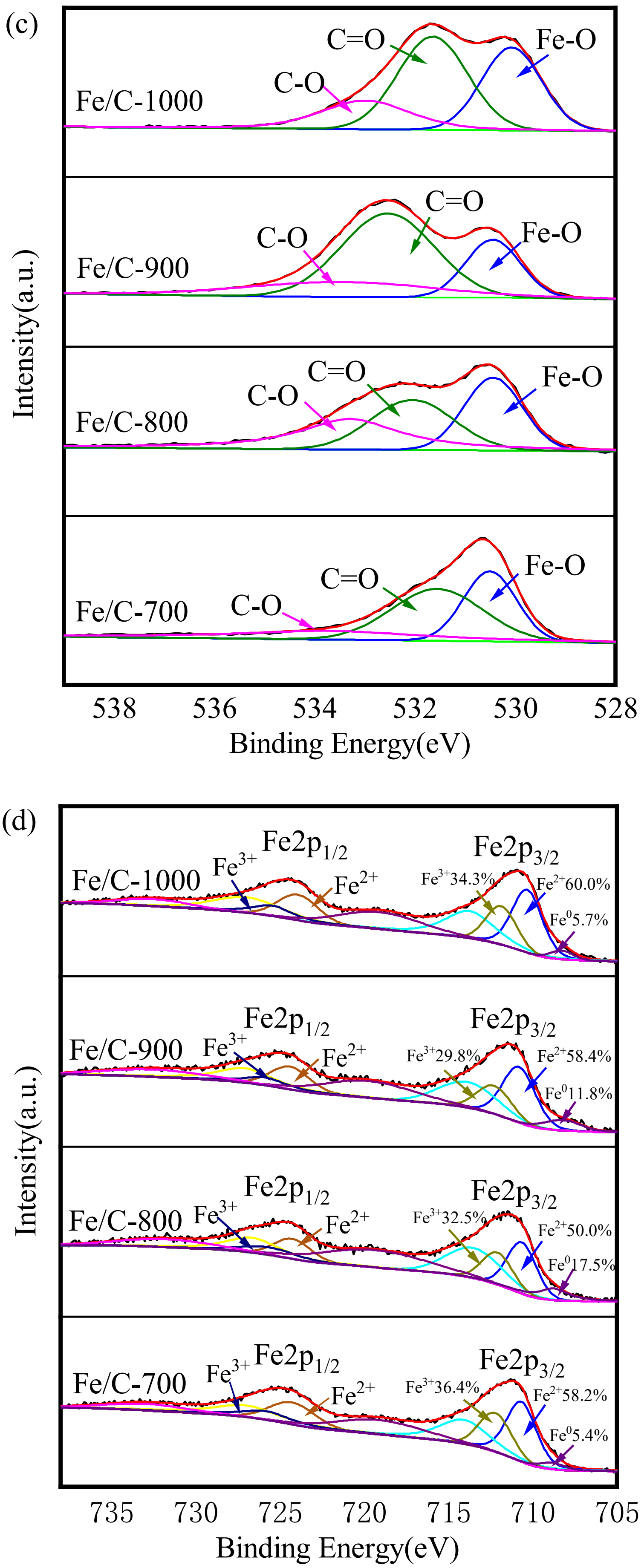

Fig. S1 XPS patterns of Fe/C catalysts: wide scan(a); C1s(b); O1s(c); and Fe2p(d)
